# Ecological Footprint Model Using the Support Vector Machine Technique

**DOI:** 10.1371/journal.pone.0030396

**Published:** 2012-01-23

**Authors:** Haibo Ma, Wenjuan Chang, Guangbai Cui

**Affiliations:** 1 College of Hydraulic & Environmental Engineering, China Three Gorges University, Yichang, China; 2 College of Hydrology and Water Resources, Hohai University, Nanjing,China; Hungarian Academy of Sciences, Hungary

## Abstract

The per capita ecological footprint (EF) is one of the most widely recognized measures of environmental sustainability. It aims to quantify the Earth's biological resources required to support human activity. In this paper, we summarize relevant previous literature, and present five factors that influence per capita EF. These factors are: National gross domestic product (GDP), urbanization (independent of economic development), distribution of income (measured by the Gini coefficient), export dependence (measured by the percentage of exports to total GDP), and service intensity (measured by the percentage of service to total GDP). A new ecological footprint model based on a support vector machine (SVM), which is a machine-learning method based on the structural risk minimization principle from statistical learning theory was conducted to calculate the per capita EF of 24 nations using data from 123 nations. The calculation accuracy was measured by average absolute error and average relative error. They were 0.004883 and 0.351078% respectively. Our results demonstrate that the EF model based on SVM has good calculation performance.

## Introduction

The ecological footprint (EF) approach was developed by Wackernagel and Rees [1]. It is calculated as the total area of bio-productive land and water required to continuously produce all resources consumed, and to assimilate all wastes generated by a defined population in a specific location [2]. The EF approach provides a comprehensive unit of measurement that allows for comparisons of various types of consumption-based impacts [3]. Therefore, since its development the EF approach has become the most widely-used measure of environmental sustainability [4].The EF approach aggregates typically complex resource use patterns into a single number [5]. The validity of the per capita EF, which traces the average amount of resources a person in a given country consumes, and the amount of waste they generate is confirmed by its significant correlation with important environmental impacts, for example, national emissions of ozone depleting substances, and nuclear power generation [6].

There are six resources considered by the EF: crop and pasture lands for production of goods and food, built land for construction, forest for the production of wood products, fossil energy for carbon dioxide emissions from fuels, and fish for food production. All of these are measured in global hectares (ha). A global hectare represents a hectare of land with global average bio-productivity. Social scientists and policymakers can compare the per capita EF of various nations to the per capita ecological capacity that exists on earth. For example, in 1996 the per capita EF ranged from 0.35 hectares to more than 16 hectares, and the majority of the estimated per capita EFs were higher than the Earth's bio-capacity per capita [7]. According to McDonald and Patterson [8], the global EF is at least 30% larger than the Earth's bio-capacity, illustrating the severity of resource overuse. EF figures can also be used as benchmarks for assessing sustainability at a national level, for example, nations with an EF at or below 1.8 hectares per capita have a global impact that could be replicated by other nations without threatening long-term sustainability [2].

Although the EF model has been used at various levels, including global [9], municipal [10], national [9], city [11] and individual [12], no previous studies have attempted to apply a support vector machine (SVM) to predict national EF. In this paper, we seek to fill this research gap by calculating the EF of 23 nations through the use of SVM techniques. The countries analyzed in this study are listed in the Appendix S1. More specially, the purpose of this research is twofold:

First, to determine the major factors influencing national EFs, and second, to build a SVM model based on these identified factors to calculate EF.

## Materials and Methods

### Materials

Drawing on previous research, we found a wealth of evidence suggesting that a variety of factors influence EF. Cross-sectional analyses consistently show that national per capita ecological footprints are largely a function of gross domestic product (GDP) [13], [14], [15]. A negative relationship between per capita EF and export dependence (measured as the proportion of total GDP generated by exports) has also been identified [15]. According to Jorgenson and Burns [16], nations with a greater intensity in the services sector experience higher increases in per capita EF.

Some evidence suggests that domestic income inequality is negatively related to the relative size of a nation's per capita EF [16]. Jorgenson (2003) found that urbanization has a positive impact on EF [13]. From the above, it can be seen that the factors that influence EF can be characterized as affluence (as measured by GDP), export dependence, service intensity, domestic income inequality, and urbanization.

### Methodology

The SVM is a machine-learning method based on the structural risk minimization principle from statistical learning theory. It maps input data 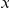 into a higher-dimensional feature space 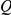 by non-linear mapping to yield and solve a linear regression problem in this feature space [17]. The regression approximation addresses the problem of estimating a function based on a given set 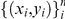, where 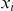 denotes the input vector, 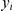 denotes the output value, and 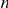 denotes the total number of data patterns. In SVM, the regression function is given as the following:
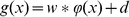
(1)Where 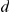 is a scalar threshold, 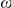 is the weight vector, and 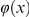 is the high-dimensional feature space that is nonlinearly mapped from the input space 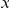.

Support vector regression (SVR) performs linear regression in the high-dimensional feature space by e-insensitive loss. At the same time, to prevent over-fitting and thereby improving the generalization capability, the following regularized functional involving summation of the empirical risk and a complexity term 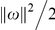, is minimized. The coefficients 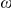 and 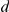 can be estimated by minimizing the regularized risk function:

Min 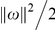


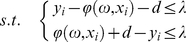
(2)


The regression problem is transformed into the following constrained formation:
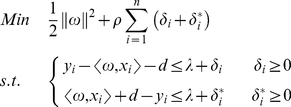
(3)Where the constant 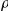 stands for the penalty degree of the sample with error exceeding 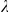. Two positive slack variables 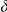 and 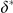 represent the distance from actual values to the corresponding boundary values of 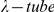.

A dual problem can then be derived by using the optimization method to maximize the function:
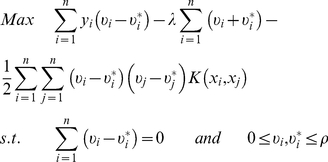
(4)Where 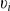 and 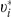 are the Lagrange multiplier.

The SVM for function fitting obtained by using the above mentioned maximization function is then given by the following function:
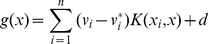
(5)In Equation 5, sample points that appear with non-zero coefficients are the so-called support vectors. The kernel function 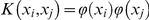 satisfies Mercer's conditions and performs the non-linear mapping.

## Results and Discussion

### Preliminary data analysis

In this study, per capita EF was taken from White [18], [19], the latest data on national level per capita EF, and GDP data were taken from the World Bank [20]. To correct for excessive skewness, we use the natural logarithm transformation of GDP data. Export data as a percentage of total GDP were taken from the World Bank [20] and used as a measure of export intensity and export dependence. Service data as a percentage of total GDP were taken from the World Bank [20] and used as an indicator of service intensity. Domestic income inequality data were taken from the World Bank [20] and are presented as GINI coefficients, which measure the distribution of income within countries. A GINI index score of zero suggests perfect equality, while an index score of 100 suggests perfect inequality. Urbanization data were taken from the World Bank [20], and are measured as the percentage of the total population living in cities, which represents a country's relative level of urbanization. Following Jorgenson and Burns [16], we regress these data on per capita GDP and use the residuals as measures of urbanization to minimize collinearity.

Table 1 provides descriptive statistics for all variables used in the analysis. The product moment correlations between variables are shown in the Table 2. Although correlations do not prove causation, they can be used to generate hypotheses; therefore Table 2 is presented to highlight the correlations among the five variables used for analysis. It indicates that most of the correlations were significant and in the expected direction.

**Table 1:** Descriptive statistics of all the variables used in this study(N = 123).

Variable	Mean	SD	Minimum	Maximum
EF	2.436	1.9517	0.52	9.59
GDP(ln)	7.618	1.6658	4.449	10.805
Service (%)	54.066	13.003	20.542	78.53
Export (%)	36.311	18.280	7.272	83.83
Gini	38.931	10.040	19.5	62.9
urban	0.441	1.053	0.001	7.296

Note: EF = ecological footprint; GDP(ln) = gross domestic product(the natural logarithm transformation); service (%) = service as the percentage of GDP; export(%) = export as the percentage of GDP. Gini = national income disequilibrium; urban = urbanization level(residualized).

**Table 2:** Product moment correlations matrix.

	1	2	3	4	5	6
1.GDP(ln)	1					
2.urban(residualized)	0.423**	1				
3.Service (%)	0.729**	0.257**	1			
4.GINI	−0.394**	−0.255**	−0.241**	1		
5.Export(%)	0.284**	0.304	0	−0.202*	1	
7.EF	0.860**	0.347**	0.613**	−0.432**	0.218*	1

**correlation is significant at the level of 0.01 (2-tailed).

*correlation is significant at the level of 0.05(2-tailed).

### SVM analysis

We used data of 123 countries (shown in Appendix S1) to establish and test the SVM-based model. We used data of 99 countries (80% of the data) to establish the model, and 24 countries (20% of the data) to test the accuracy of the model. For the 24 countries used to test model accuracy, the calculations were conducted in alphabetical order; therefore the results are presented alphabetically. When the correct model was established, there was no need for further “training” or “test” data. The model only required five variables to calculate a nation's EF. In addition, the model was designed to achieve short calculation times. Therefore, compared to traditional EF techniques, the SVM technique was very easy to apply.

**Figure 1:**
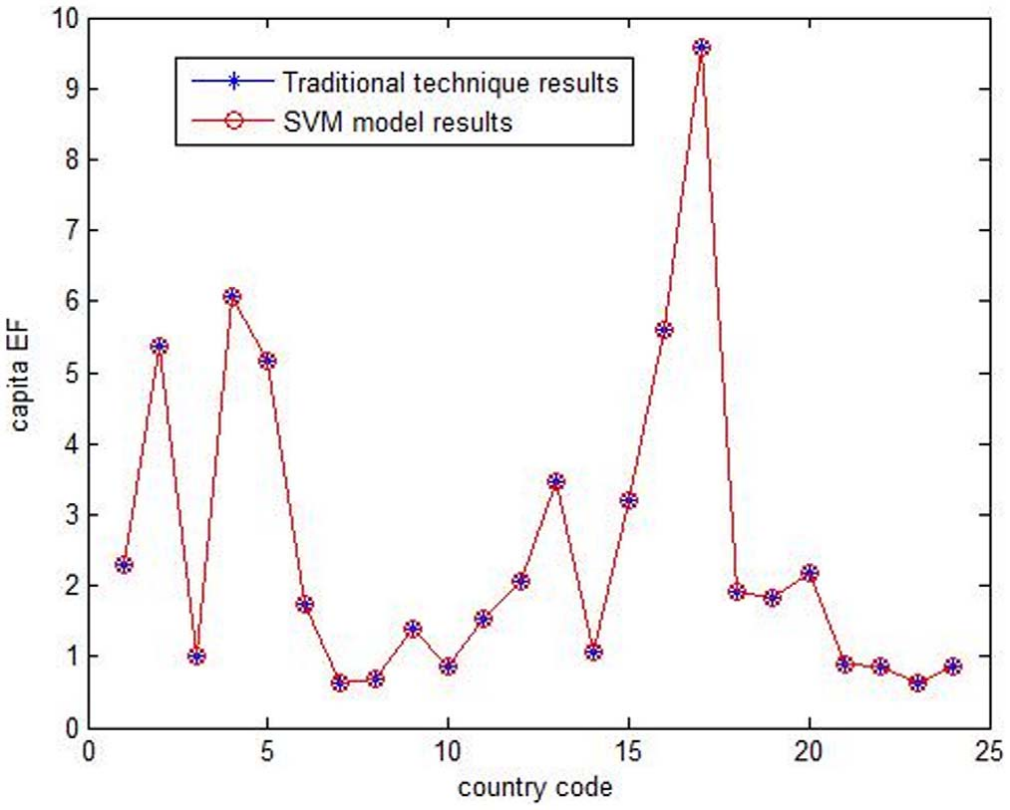
SVM calculation performance of per capita EF.

According to the method of Liu, Zhuang, and Liu [17], we used the particle swarm optimization technique to choose the optimal parameters for the SVM model. The optimal parameters are as follows: 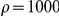, 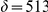, 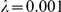. The EF model was then determined by these three parameters and the data of 99 countries. Following this, we used the model to calculate the EF of the other 24 countries. Model accuracy was measured by absolute and relative error. The calculation performance is displayed in Figure 1. The calculation results are presented in Table 3. Figure 1 and Table 3 show that the EF model based on SVM can calculate EF perfectly. The average absolute error is 0.004883, and the average relative error is only 0.351078%. Therefore, we were successful in establishing an EF model, and we can use it to calculate the EF of any nation using only five nation-specific variables.

**Table 3:** Calculation results by SVM.

Country name	Traditional technique results	SVM model results	Absolute error	Relative error(%)
South Africa	2.29	2.2946	0.0046	0.200873
Spain	5.36	5.3551	0.0049	0.09142
Sri lank	1	1.0046	0.0046	0.46
Sweden	6.07	6.0664	0.0036	0.05931
Switzerland	5.15	5.1442	0.0058	0.11262
Syria	1.73	1.7364	0.0064	0.369942
Tajikistan	0.64	0.6442	0.0042	0.65625
Tanzania	0.7	0.7069	0.0069	0.985714
Thailand	1.38	1.3866	0.0066	0.478261
Togo	0.87	0.8742	0.0042	0.482759
Tunisa	1.54	1.5469	0.0069	0.448052
Turkey	2.06	2.0629	0.0029	0.140777
Turkmenistan	3.47	3.4666	0.0034	0.09798
Uganda	1.08	1.0853	0.0053	0.490741
Ukraine	3.19	3.1847	0.0053	0.16614
United kingdom	5.59	5.5842	0.0058	0.10376
United states	9.59	9.5843	0.0057	0.05944
Uruguay	1.92	1.9231	0.0031	0.161458
Uzbekistan	1.83	1.8346	0.0046	0.251366
Venezuela	2.18	2.1832	0.0032	0.146789
Vietnam	0.88	0.8846	0.0046	0.522727
Yemen	0.85	0.8542	0.0042	0.494118
Zambia	0.63	0.6354	0.0054	0.857143
zimbabwe	0.85	0.8550	0.005	0.588235
average	2.535417	2.537425	0.004883	0.351078

According to Table 2, we can see that the product moment correlation between GDP (ln) and EF was 0.860. We constructed a least-squares regression model and obtained the following equation:
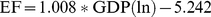
(6)The average absolute error from least-squares regression is 0.7620, and the average relative error is 44.66%. These are bigger than the errors derived from the EF model using the SVM technique with five variables. So, we can assume that the additional four variables are useful for the calculation of EF.

### Implications, limitations, and future research

Our results demonstrate that national level per capita EF is influenced by the nation's GDP, urbanization level, distribution of income (measured with the Gini coefficient), export dependency (as a percentage of total GDP), and service intensity (as a percentage of total GDP). Using these five variables, we established an SVM model to calculate EF. Compared with the traditional technique, the SVM model required less variables, and had a quicker calculation time. Therefore, the SVM technique is very easy to apply.

Despite the significant contributions of this study, it is subject to a number of limitations. First, this study used a cross-sectional rather than a longitudinal method. Much more emphasis was placed on observing national-level EFs than on observing changes in global EF. Much more emphasis should be placed on longitudinal research to focus on observing changes in EF behavior over time. Second, we only considered five factors that influenced per capita EF. In the future, we will explore other factors influencing per capita EF.

As a new approach to measuring sustainability, EF analysis has been more successful than others. Inevitably, the approach is not without its flaws [21], [22]. However, its theory and application will be improved with continued study and with refinements the methodology used by organizations responsible for environmental reporting and management.

## Supporting Information

Appendix S1 **Countries analyzed in the study.**
(DOC)
