# Correction: Ecological Footprint Model Using the Support Vector Machine Technique

**DOI:** 10.1371/annotation/cf7186cf-134b-49e8-b6be-b3e48e694eca

**Published:** 2012-02-29

**Authors:** Haibo Ma, Wenjuan Chang, Guangbai Cui

There was an error in the Funding statement. The correct Funding statement is, "This work was partly supported by the special fund for public scientific research from the Ministry of Water Resources of China (NO.201001003) http://www.chinawater.net.cn/zt/07gyxky/. [^] The funders have no role in study design, data collection and any analysis, decision to publish, or preparation of the manuscript." 

