# Supplementary material for: Ecological Footprint Model Using the Support Vector Machine Technique
Source: PLoS One. 2012 Jan 23;7(1):e30396. doi: 10.1371/journal.pone.0030396 (PMC3264588; doi:10.1371/journal.pone.0030396)
Supplement: Appendix S1 — Countries analyzed in the study. (DOC) [file pone.0030396.s001.doc]

**Appendix S1. Countries analyzed in the study**

| Albania | Czech republic | Panama | Ireland |
| --- | --- | --- | --- |
| Algeria | Denmark | Paraguay | Italy |
| Angola | Dominican republic | Peru | Japan |
| Argentina | Ecuador | Philippines | Jordan |
| Armenia | Egypt | Poland | Kazakhstan |
| Australia | E1 Salvador | Portugal | Kenya |
| Austria | Eritrea | Romania | Korea republic |
| Azerbaijan | Estonia | Russia | Kuwait |
| Bangladesh | Ethiopia | Rwanda | Kyrgyzstan |
| Belarus | Finland | Saudi Arabia | Laos |
| Belgium | Lithuania | Senegal | Switzerland |
| Benin | Macedonia | Sierra Leone | Syria |
| Bolivia | Madagascar | Slovakia | Tajikistan |
| Botswana | Malawi | Slovenia | Tanzania |
| Brazil | Malaysia | South Africa | Thailand |
| Bulgaria | Mali | Spain | Togo |
| Burkina Faso | Mauritania | Sri lank | Tunisia |
| Burundi | Mexico | Sweden | Turkey |
| Cambodia | Moldova republic | Latvia | Turkmenistan |
| Cameroon | Mongolia | Lebanon | Uganda |
| Canada | Morocco | France | Ukraine |
| Central African rep | Mozambique | Germany | United kingdom |
| Chad | Namibia | Ghana | United states |
| Chile | Nepal | Greece | Uruguay |
| China | Netherlands | Guatemala | Uzbekistan |
| Colombia | New Zealand | Guinea | Venezuela |
| Congo | Nicaragua | Honduras | Vietnam |
| Congo, Dem. Rep | Niger | Hungary | Yemen |
| Costa Rica | Nigeria | India | Zambia |
| Cote d’Ivoire | Norway | Indonesia | Zimbabwe |
| Croatia | Pakistan | Iran |  |
